# Horses’ Use of Lying Halls and Time Budget in Relation to Available Lying Area

**DOI:** 10.3390/ani11113214

**Published:** 2021-11-10

**Authors:** Linda Kjellberg, Jenny Yngvesson, Hanna Sassner, Karin Morgan

**Affiliations:** 1 Swedish National Equestrian Center Ridskolan Strömsholm, Stallbacken 6, SE-734 94 Strömsholm, Sweden; karin.morgan@rsflyinge.se; 2 Department of Anatomy, Physiology and Biochemistry, Swedish University of Agricultural Sciences, Box 7011, SE-750 07 Uppsala, Sweden; 3 Department of Animal Environment & Health, Swedish University of Agricultural Sciences, Box 234, SE-532 23 Skara, Sweden; jenny.yngvesson@slu.se; 4 Department of Biosystems and Technology, Swedish University of Agricultural Sciences, Box 190, SE-234 22 Lomma, Sweden; hanna.sassner@slu.se; 5 Swedish National Equestrian Center Flyinge, Flyinge Kungsgård, SE-247 93 Flyinge, Sweden

**Keywords:** lying time, recumbency, group housing, lying behavior, shelter, open barn system, sleep, rest, welfare

## Abstract

**Simple Summary:**

A safe and comfortable resting area to lie down and sleep in is an important factor in ensuring horse welfare. The lying times of stalled horses depend on factors such as bedding, housing, and lying area, while the sleeping behavior of group-housed horses may be influenced by such factors as social relations and competition for space. This study aimed to analyze time spent in, as well as activity taking place in, lying halls of various sizes. We compared single boxes and open barns with available lying areas of 8, 15, or 18 m^2^/horse, on the basis that a lying area of 8 m^2^ is the minimum requirement stipulated by Swedish legislation. We found that increasing lying area increased the horses’ use of the lying hall and their total lying time, and that the lying time of a horse housed in a single box was equivalent to the lying time of a horse in group housing with access to a lying area of 18 m^2^/horse. Hence, to ensure access to sufficient resting space for all horses in group housing, we recommend that the minimum requirement should be reassessed and increased.

**Abstract:**

Sleep is crucial to horses’ wellbeing, and their lying time can vary according to such factors as climate, exercise, bedding, and housing. This study aimed to analyze behavior and time spent in lying halls of various sizes. We examined the influence of housing systems on total lying time and behavior, and how changes to available lying area can affect lying time. Two open barns were used in this study, with lying areas of 8, 15, and 18 m^2^/horse available in the lying halls. The horses’ behavior was video recorded and logged using scan sampling and interval observations. Individual boxes were used as a control. The horses were found to spend longer time in sternal and lateral recumbency in the hall with a lying area of 18 m^2^/horse than the hall with a lying area of 8 m^2^/horse. Increasing the area of the lying hall also increased overall time spent there. Consequently, the hypothesis that increasing lying area will increase the horses’ use of the lying hall, as well as their total lying time, was accepted.

## 1. Introduction

Ensuring horse welfare is vital for riders and owners alike, both for ethical reasons and to ensure their health and happiness. From an ethical perspective, it may be argued that because we use horses for sport and leisure, we have a responsibility to protect their wellbeing, not only in preventing suffering, but also in promoting positive emotional states, as Mellor & Burns [1] concluded. The theoretical framework of animal welfare has been revised in 2015 by Mellor & Beausoleil [2] to include five domains (nutrition, environment, health, behavior, and mental state) and emphasize the importance of positive mental states. Furthermore, Mellor & Burns [1] have developed a practical assessment model for equine welfare, within which the present study is particularly concerned with the domains of Environment and Behavior. Housing horses in an open barn system may substantially benefit their welfare, as it gives them more choice, allows access to a larger space, and provides the opportunity to move around at all times. Yngvesson et al. [3] found that horses in Swedish riding schools were in better general health when kept in group housing then when they were housed individually and they had a lower incidence of respiratory problems and colic. Additionally, the open barn system is conducive to an improved working environment, as difficult and time-consuming tasks can be mechanized. The basic design consists of a lying hall, where the horses are provided with a dry and comfortable lying surface, and a paddock around the lying hall, with forage delivered ad lib or by computer-aided feeding stations. However, open barns can differ greatly in their planning and management. More research data are thus required to develop guidelines for suitable layouts, ensuring the sustainability of the horses’ welfare.

An important factor in the welfare of horses is the opportunity to get enough sleep. Horses move through four stages of vigilance: wakefulness, drowsiness, slow wave sleep, and paradoxical sleep [4,5,6]. Even though horses can sleep while standing, they also need to lie down and rest their heads, which happens during paradoxical sleep. Monitoring the horses’ lying time can be used to compare different housing systems from a welfare perspective [7]. It has been observed that feral horses tend to spend 0.5–2 h lying down during a 24 h period [8,9,10,11], and foals and young horses (2–3 years old) tend to spend even longer [9]. Individually stalled horses usually spend 3–5 h lying down during a 24 h period [4,5,12], while lying time for horses kept in open barns reportedly ranges from 1 to 2 h [13,14]. Raspa et al. [15] suggested that the longer lying times found in stalled young horses compared to feral young horses could be due to boredom and restricted space. Using an automatic concentrate station increased lying time from 84 ± 42 min to 115 ± 71 min per day [14]. In the same study, they found that the lying periods in this active open barn seemed to occur between the hay feeding times. 

Total lying time can vary according to several factors. It tends to be shorter, for example, in hot climates [16], and longer with increased exercise [17]. Köster et al. [12] found that horses kept in open barns had shorter lying times than those kept in individual boxes, and horses kept in open barns were said to have similar lying times [13,14,18] to feral horses [8,10,11]. Studies also show that as many as one-third of horses kept in active open barns do not lie down every day [19]. Lower-ranked horses in open barns spend little to no time lying down when compared with higher-ranked horses [19,20,21], especially when the lying area is restricted [21]. Lower-ranked horses reportedly exhibit fewer lying bouts than higher-ranked horses [19] because their lying bouts are often disturbed or interrupted [20]. In comparing the activity levels of stabled horses and horses kept in open barns, Gansow [22] found a negative correlation between activity level and rank for the horses kept in open barns.

Fader & Sambraus [21] found that the size of the lying area influenced time spent lying down. Horses in open barns with a smaller lying area (4.6 m^2^/horse, 59 ± 48 min) spent significantly less time lying down (*p* < 0.01) than horses with access to a larger lying area (10.0 m^2^/horse, 103 ± 73 min, 17.3 m^2^ per horse, 134 ± 37 min). In two of the open barns, the lying area was divided between two lying halls, and the lying time for those herds did not differ significantly from the other herds. Moreover, Raabymagle & Ladewig [23] found that lying time was shorter in smaller boxes ((1.5 times withers’ height of the horse)^2^ m^2^) compared with larger boxes ((2.5 times withers’ height of the horse)^2^ m^2^). In an open barn housed by broodmares, the proportion of the herd using the lying hall increased when the number of horses was reduced, which basically increased the available lying area from 7 to 17 m^2^ [24]. The same study found that the mares used the lying hall in turns at a higher occupancy rate, suggesting that they divided themselves into subgroups. Swedish legislation for animal welfare stipulates that the minimum lying area in open barns must be 8 m^2^/horse for large horses (determined by height of the withers over 1.71 m) when they are being fed outside the lying hall, and 10 m^2^/horse when they are being fed inside [25]. Besides lying area, bedding material seems to be another factor that influences lying time, both in boxes and open barns. Extending the area covered by soft bedding materials, such as straw or wood shavings, increased both lying time and duration [20]. Lying times in an active open barn were found to be longest for shavings (74.3 ± 2.87 min), followed by rubber mats (62.3 ± 2.27 min), and then sand (43.0 ± 2.33 min) [19]. Using rubber mats instead of wood shavings in single boxes led to shorter lying times [26]. Werhahn et al. [27] found that foraging increased when straw bedding was used, indicating that this kind of bedding poses the risk of interrupting lying bouts. 

Furthermore, horses’ use of lying halls seems to be determined by the weather, and wet and windy conditions have been shown to increase the use of lying halls [28,29,30]. Feral horses also exhibit this behavior, seeking both shade and shelter from insect harassment [31], as well as protection from the wind and rain [32]. Individual spacing area is another factor that could influence the use of lying halls. Keiper & Sambraus [33] found that stallions spend most of their time with 1 m to 10 m between themselves and other horses. Mares tended to require less individual space, because they were broodmares with offspring nearby. Differences in individual spacing area, where foals were found to be more willing to share space than older horses [30], indicate that the adult horse’s individual space requirements are likely to be >1 m.

On this basis, we believe there is demand for research into the “best practice” for planning and managing animal welfare in open barns, which should not be dictated solely by minimum requirements. Sleep is an important factor in the welfare of horses and lying time for stalled horses seems to vary according to such factors as climate, exercise, bedding, housing, and lying area. To ensure the wellbeing of the horses in this type of housing system, it is critical that lying areas provide the opportunity for adequate rest and sleep. 

The aim of this study was to analyze time spent in, as well as activity taking place in, lying halls of varying sizes. The study had three research questions: How does the use of lying halls depend on available lying area? In what way does the lying time differ in individual boxes compared to shared lying area in an open barn? How do variations in total available lying area affect lying time? The hypothesis was that increasing available lying area would increase total lying time. 

## 2. Materials and Methods

To answer the questions, we designed two different studies performed at two of the Swedish National Equestrian Centers: Flyinge (Study 1) and Strömsholm (Study 2). In both stables, the horses were housed in an open barn system for 24 horses and fed using the feeding technique from HIT Active Stable^®^ (Weddingstadt, Germany). Within this system, haylage and concentrate were delivered by automatic computer-controlled feeding stations, as described by Kjellberg & Morgan [34]. The feeding stations were set to start a new feeding session at 8 AM in the active open barn at Flyinge and at 6 AM in the active open barn at Strömsholm. Straw was delivered ad lib in one hayrack, and the bedding in the lying halls was straw, so the horses were able to feed there as well. The system also made use of automatic watering balls. 

### 2.1. Study 1—Open Barn at National Equestrian Center Flyinge

#### 2.1.1. Horses

All 18 geldings, aged 6–21 years, including five focal horses, aged 10–16 years, were school horses at the equine center, used for riding or driving at the university equine bachelor program or the upper secondary school. They were all Swedish Warmblood, except for one horse which was a North Swedish draft horse. The horses were trained 5 days/week at an intensity dictated by their education, with 1 day/week spent hacking. All horses were well accustomed to each housing system and had spent at least two months in the actual open barn before the study.

#### 2.1.2. Facilities

**Figure 1:**
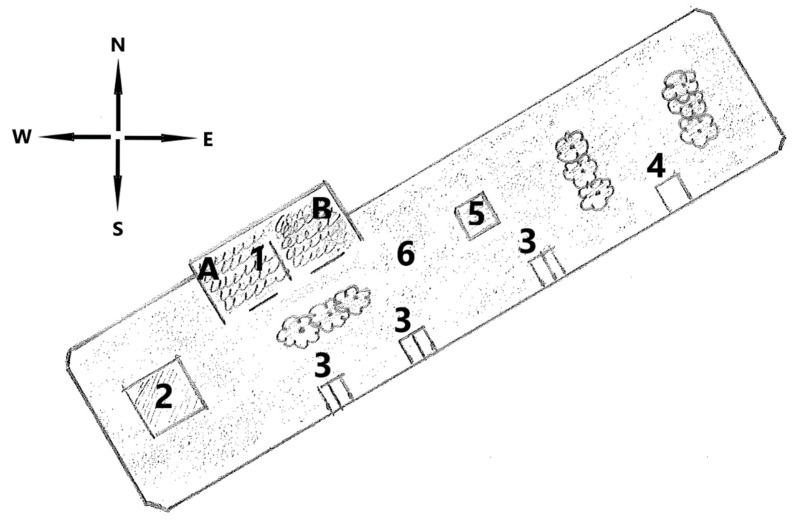
Detailed layout of the open barn at Flyinge in Study 1. 1: Lying hall, 2: roller pit, 3: automatic forage stations, 4: concentrate station, 5. hay bar, 6: paddock, A: camera 1, B: camera 2.

The open barn system consisted of one paddock of 3500 m^2^ and one lying hall with a lying area of 280 m^2^ (Figure 1). Haylage was delivered by six automatic forage feeding stations, and concentrate by one automatic concentrate feeding station. The system also contained three automatic watering balls and one roller pit. The horses’ use of, and behavior in, the lying hall were monitored using up to two IR night-and-day network cameras for outdoor use.

#### 2.1.3. Data Collection 

**Figure 2:**
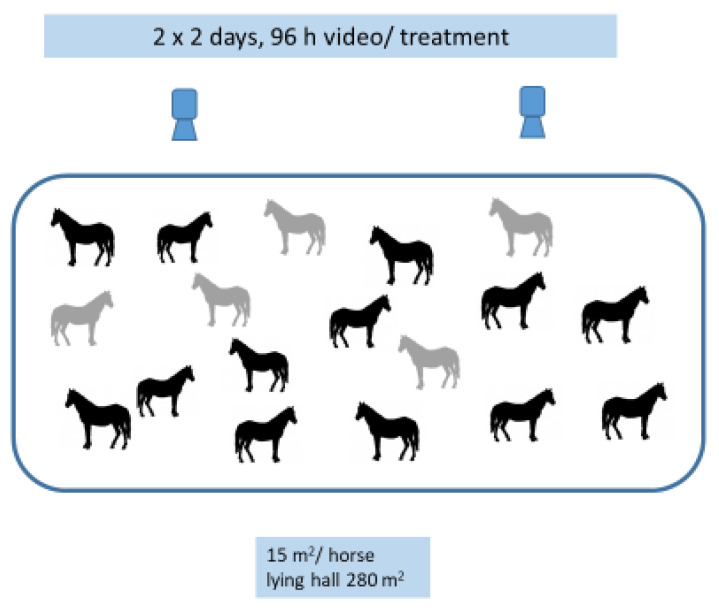
Illustration of the two methods used in the data collection for Study 1. The grey horses (*n* = 5) were used in method 1 (interval registrations each minute) and all horses (*n* = 18) were used in method 2 (scan sampling every hour).

Two methods were used for data collection in the lying area (Figure 2). In method 1, the video recording was continuous, but all observations were logged as interval observations using an ethogram (Table 1), for each minute that the five randomly chosen focal horses spent in the lying hall. The horses used in method 1 were not allowed to leave the system during the two weekends when data were collected. Method 2 used scan sampling, recording the positions of all 18 horses in the lying hall every hour. No video recordings were conducted outside the lying hall. The video recording was performed over two weekends at the end of October and beginning of November. The temperature daytime shifted from +12 °C to +14 °C and from +2 °C to +8 °C at night. Data were collected in collaboration with students from the university equine bachelor program SLU and preliminary analysis was presented in Bengtsson & Eriksson [35].

#### 2.1.4. Statistical Analyses

The data collected in Study 1 were compiled using Microsoft Excel. We calculated the mean, maximum and minimum values, as parameters for lying periods (Method 1) and the number of visits to the lying hall (Method 1 and 2). The time budget was calculated for the five focal horses (Method 1). The proportion of observations of the horses’ locations (inside or outside the lying hall) was calculated both for the entire day and for day or nighttime. The difference between day and night was analyzed using a Student’s t-test. The significance level was set to *p* < 0.05.

### 2.2. Study 2—Open Barn at National Equestrian Center Strömsholm

#### 2.2.1. Horses

All 11 geldings, aged 3–17 years, were Swedish Warmblood and were used as riding horses at the university equine bachelor program. The horses were trained 3–4 days/week at an intensity dictated by their education, with 2 days/week spent hacking, except for the 3-year-old. The horses were 1.62–1.74 m over the withers in height. All horses were well accustomed to each housing system and had spent at least two months in the actual open barn before the study.

#### 2.2.2. Facilities

A stable with 25 single boxes, each with an area of 10.5 m^2^ (3 × 3.5 m) was used as a control. This stable was situated immediately next to the active open barn. When the horses were housed in the single boxes, they were fed forage individually four times a day, and concentrate twice. They spent 4–6 h daily in a paddock. Each single box had an automatic watering ball and was permanently bedded with wood shavings. The horses could socialize with their neighboring horses through nose contact via grids.

**Figure 3:**
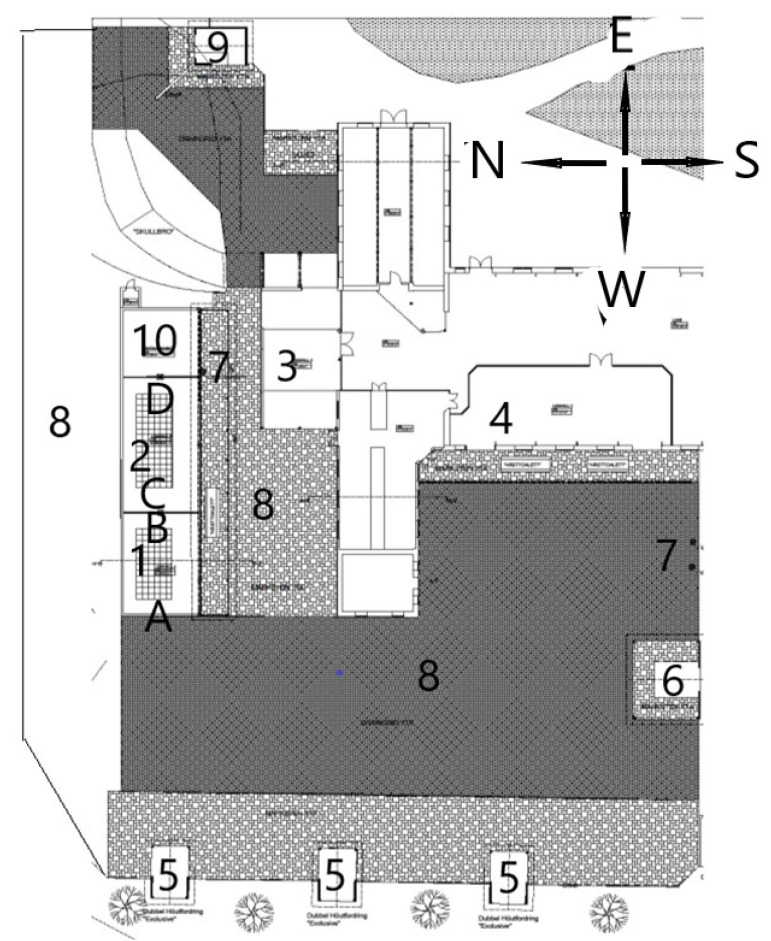
Detailed layout of the open barn. 1: Lying hall 1 (80 m^2^, used in period 2 and 3), 2: Lying hall 2 (100 m^2^, used in period 3), 3: Lying hall 3 (100 m^2^, not in use during experimental study), 4: Lying hall 4 (280 m^2^, not in use during experimental study), 5: automatic forage stations, 6: straw bar (not in use), 7: watering balls, 8: paddock, 9: concentrate station, 10: acclimatization box, A: camera 1, B: camera 2, C: camera 3, D: camera 4.

The open barn system consisted of one paddock of 3600 m^2^ and had a total indoor lying area of 460 m^2^ divided into four lying halls, with the possibility of closing individual lying halls (Figure 3). Haylage was delivered by six automatic forage feeding stations, and concentrate by one automatic concentrate feeding station. The system also contained three automatic watering balls. The horses’ use of, and behavior in, the lying halls were monitored during three periods, using up to four IR night-and-day network cameras for outdoor use.

#### 2.2.3. Data Collection

**Figure 4:**
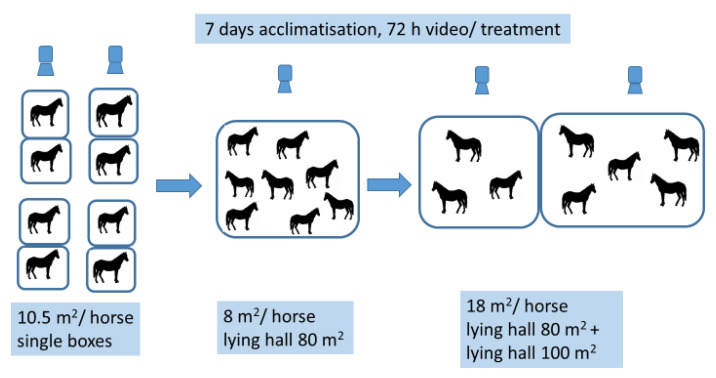
Flowchart illustrating the data collection during the three periods. Eight individuals participated in all three periods. These eight individuals were included in the herd of ten horses during period 2 and 3 in the open barn.

Each period consisted of ten days, divided into seven days of acclimatization followed by three days of video recording (Figure 4). The first period was divided in two periods, 1a and 1b, due to limited access to single boxes. In this period the horses’ lying and resting time in single boxes (10.5 m^2^) was registered, before moving back to the open barn with its different available lying areas in the lying halls during periods 2 and 3. In period 2, the horses only had access to Lying hall 1 with an available lying area of 8 m^2^/horse. In period 3, they had access to two lying halls (Lying hall 1 and Lying hall 2), with available lying areas of 18 m^2^ per horse. In periods 2 and 3, there were always ten horses housed in the open barn. In total, eleven horses were recorded using video observation across all three periods, but only eight horses participated in all three periods. Consequently, these eight horses were included in the statistics. The video recording was continuous, but all observations were logged with scan sampling, in 5 min blocks based on the ethogram (Table 1). The same ethogram used in Study 1 was used in Study 2, with some activities divided into two; “Standing attentive” was divided into “Standing attentive” or “Foraging”. “Active” was divided into “Walking” or “Other”. The study took place from the beginning of February until the middle of April 2016. The temperature in daytime during period 2 shifted from +5 °C to +15 °C and from −2 °C to +4 °C at night. During period 3 the temperature shifted in daytime from +5 °C to +8 °C and from −1 °C to 0 °C at night.

Activities in single boxes and in lying halls were monitored using up to four IR night-and-day network cameras for outdoor use (HIKVision, Hangzhou, China, model: DS 2CD4D26FWD-IZS). No video recordings were conducted in the paddock when the horses were housed in single boxes in period 1 or the lying halls in period 2 or 3. Four cameras were used during period 1a and 1b, with one camera filming each single box. Two cameras were used during period 2 for video recording Lying hall 1, and four cameras during period 3 for video recording Lying halls 1 and 2, two in each lying hall.

#### 2.2.4. Statistical Analyses

The results of Study 2 are based on mean values, from observations which took place during 72 h of video recording in period 1 and 2. There were only 48 h of video recording in period 3, as the horses tore down the barriers to a closed lying hall on the last night. Statistical analyses were conducted using RStudio (Boston, MA, USA, version 1.2.5033). The data were processed using a Poisson regression with horse as the variable factor and treatment as the fixed factor, using model: glmer1<-glmer (LieS~beh + (1|Namn), data = hast, family = “poisson”). To ensure that the variance was not the same as the mean and the analysis, a negative-binomial distribution was made according to the model: glmer.nb1<-glmer.nb (LieS~beh + (1|Namn), data = hast).

## 3. Results

### 3.1. Study 1

#### 3.1.1. Time Spent in Lying Hall

The five focal horses used in method 1 spent an average of 22% (ranging from 19.5 to 24.5%) of each 24 h period in the lying hall (Supplementary material Table S1: Rawdata Study 1—Rec. obs. focal horses). The highest recorded use of the lying hall for one horse during a 24 h period was 35.2%, and the lowest was 6.6%. The focal horses spent an average of 26% of their time in the lying hall during the night (18.00–07.59), compared with 16% during the day (08.00–17.59) (*p* = 0.004). The horses visited the lying hall an average of 5.7 (ranging from 3.6 to 7.0) times every 24 h. 

**Figure 5:**
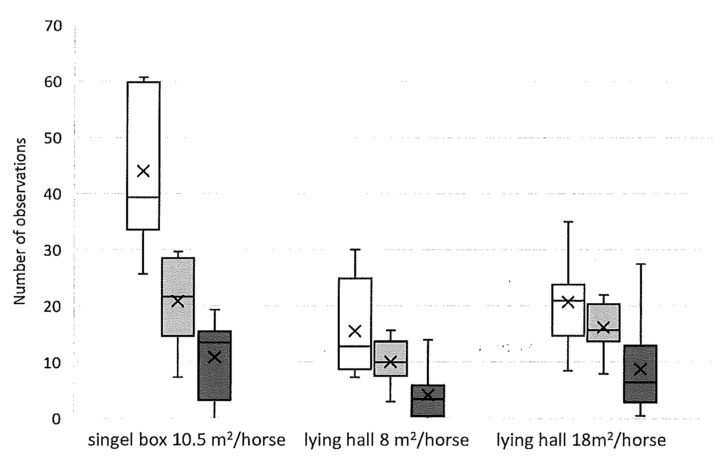
Median values for the resting observations of the horses in Study 2 (*n* = 8), for different housing systems and different available lying areas. White boxes show standing rest, light grey boxes show sternal recumbency, and dark grey boxes show lateral recumbency. The horses exhibited longer standing rest in single boxes than in the lying halls. They spent longer in both sternal and lateral recumbency in single boxes and in the lying hall with a lying area of 18 m^2^/horse compared with the lying hall with a lying area of 8 m^2^/horse.

Using hourly scan sampling for all 18 horses (method 2), the average number of horses in the lying hall was 4.1 ± 1.6 (Figure 5). The lying hall was least frequented in the morning, at 9 AM, and most frequented at night, at 2 AM, when 11 out of 18 horses spent time there. They visited the lying hall more frequently during the night than the day (*p* < 0.001). The lying hall was never empty (Supplementary material Table S1: Rawdata Study 1—No of horses in lying hall). It was always occupied by at least two horses.

#### 3.1.2. Lying Time and Time Budget

The five focal horses exhibited the following behaviors: sternal recumbency (18%), lateral recumbency (8%), standing rest (45%), standing attentive (25%), and active (4%) during the time spent in the lying hall. 

### 3.2. Study 2

#### 3.2.1. Time Spent in Lying Hall

In Study 2, in 14% of observations the horses were found in the lying hall when the available lying area was 8 m^2^/horse, compared to 33% of observations when the available lying area was 18 m^2^/horse (*p* < 0.0001) (Supplementary material Table S2: Rawdata Study 2—Recorded. obs. per horse). In period 3, when the horses had access to two lying halls with available lying areas of 80 m^2^ and 100 m^2^, respectively, the horses were observed to spend more time (76%) in the larger lying hall (100 m^2^). 

When comparing the use of the lying halls, the results showed that the lying hall with the smallest available lying area (8 m^2^/horse) was least frequented at 8 AM, 13–14 PM and 17–18 PM, and most frequented in the early morning, at 3 AM (Supplementary material Table S2: Rawdata Study 2—Use of lying hall). When the largest lying area of 18 m^2^/horse was available, the two lying halls were least frequented at 9 AM, 13 PM, and 17 PM, and most frequented at 16 PM and 2 AM. The average number of horses in the smaller available lying area of 8 m^2^/horse was 1.5 ± 1.1, and in the larger available lying area of 18 m^2^/horse it was 2.9 ± 2.5. The highest number of horses observed in the lying halls was eight horses out of ten when the available lying area was 8 m^2^/horse and nine horses out of ten when the available area was 18 m^2^/horse. 

#### 3.2.2. Lying Time and Time Budget

The horses’ lying times for both sternal and lateral recumbency were significantly lower in the smaller lying hall with an available lying area of 8 m^2^/horse versus 18 m^2^/horse (*p* = 0.001 and 0.02, respectively) and the single boxes (*p* ≤ 0.0001 and 0.0002, respectively) (Figure 5). Standing rest was longer in single boxes than in the lying halls with available lying areas per horse of 8 m^2^ and 18 m^2^ (*p* ≤ 0.0001). There was no difference in total resting time (sternal and lateral recumbency and standing rest) between the lying halls with different lying areas per horse (8 m^2^ and 18 m^2^). Moreover, none of the horses were seen lying down in the paddock during the period in which they were housed in single boxes.

**Figure 6:**
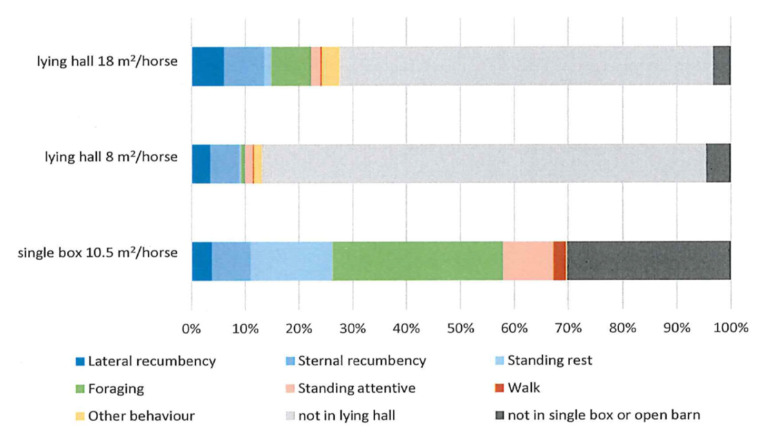
The chart shows the time budgets for single boxes and active open barn with lying areas of 8 m^2^ and 18 m^2^ in Study 2 in Strömsholm based on 10 horses.

In comparing activity in the lying halls, we can see that the horses spent more time foraging from the bedding when they had access to a lying area of 18 m^2^/horse than when they had access to an area of 8 m^2^/horse (*p* < 0.0001) (Figure 6). There were no differences in standing attentive or other behaviors between the lying halls with different available lying areas. They did exhibit other behaviors in the lying halls, as compared with the single boxes, such as play. The horses also used the lying halls for urination. They were also standing attentive, which included foraging, in 8% of the observations. In the single boxes, the behavior of the horse was restricted during some observations by human interaction, when the horse was tied up while their box was cleaned, or handled by the groom in other ways.

## 4. Discussion

Housing horses in an open barn system may substantially improve their welfare, as Yngvesson et al. [3] found, provided that the horse’s nutritional needs are also met and that they are part of a suitable social group where competition for lying space is low. Ensuring their welfare does not only mean preventing suffering, but also promoting positive emotional states [1]. The domains Environment & Behavior are particularly relevant in open barns when the horse has more choices [2]. The aim of this study was to analyze time spent, as well as activity taking place in, lying halls of various sizes. To answer the questions following the aim, we used two different studies. In the first study we received an answer for the use of and lying time in a lying hall. To follow up these findings we continued with the second study to analyze if the use of lying halls and lying time depended on available area. In the second study we also compared lying time in solitary and shared spaces. The lying area of 8 m^2^/horse in Study 2 was chosen based on the minimum requirement for a horse of a particular size stipulated by Swedish legislation [25]. The minimum requirement for a lying area in a lying hall is that it should be no less than 80% of the area of a single box which houses a horse of the same size, if the feeding area is placed outside the lying hall. The area requirement is calculated from the height of the withers, and does not consider the horse’s behaviors or need for individual space. 

The highest number of horses was found during the night (1–4 AM) in all lying halls, indicating that horses will seek shelter during nighttime whatever the available lying area, as seen in another study [27]. Since our studies were conducted during late autumn and early spring, there was a drop in temperature overnight, suggesting that the horses were seeking shelter due to weather conditions [28,29,30,32]. 

There was a wide range of time spent in the lying hall among the focal horses in Study 1, ranging from 6.6% to 35.2% of their time, during separate 24 h periods. This variation in use of lying halls is consistent with other studies and can vary due to weather or feed availability [30]. The lying halls were all facing either southeast (Study 1) or southwest (Study 2), meaning that the radiation of the sun was similar. Lying hall 1 in Study 2 (Figure 3) is less shaded by other buildings during afternoons compared to Lying hall 2 and some horses have been seen to stand there in on sunny days during the winter. The feeding stations restarted at 8 AM (Study 1) and 6 AM (Study 2) and this could explain the drop in horses each morning shortly before the restart. The same tendency for when lying periods occurred has been observed in other active open barns [13], meaning that the routines of the housing system could have influenced the lying periods. In Study 1, consisting of 18 horses and 1 lying hall, the lying hall was never empty. This finding indicated that a larger herd divides into subgroups and visits the lying hall in turns, as Nilsson [24] suggested.

In Study 2, the horses’ use of the lying hall increased from 14% to 33% when the available area increased. In comparing time spent in the two lying halls during period 3, we find that the horses preferred the larger lying hall (100 m^2^) to the smaller one (80 m^2^). This indicated that even a small increase in the lying area had an impact on the time spent in the lying hall. Although most of the horses spent all or nearly all of their time in the larger lying hall, some individuals preferred the smaller hall. In opting for the smaller hall, these horses had access to a greater lying area for themselves, instead of being squeezed in with the other horses in the lying hall next door. There could be several reasons for this choice. Some studies have found that a larger lying area leads to more frequent lying down [21,23]. Other studies have found that low-ranked horses are more frequently disturbed [20], which might also have encouraged some horses to choose the less crowded larger lying hall. Individual spacing from other horses often depends on sex or age [30,33]. In Study 2, one horse was seen lying outside the lying hall during daytime when the horses had access only to the smallest available lying area (8 m^2^). Consequently, we can speculate that this horse might have found it too crowded in the lying hall and required more individual space even though neither individual spacing nor rank was measured in this study.

In Study 2 we found that the horses demonstrated longer estimated sternal and lateral recumbency in the single boxes (10.5 m^2^) and in the lying halls with a larger available lying area (18 m^2^/horse), than they did in the lying halls with a smaller available lying area (8 m^2^/horse). Still, the estimated average lying time in the single boxes was slightly lower (2 h and 40 min) than the length of 3 to 4 h reported in other studies [4,5,12]. The lying time in single boxes was still longer than the 30 min to 2 h reported for feral horses [8,9,10,11], suggesting that the horses in single boxes obtained more lying time than horses in more natural circumstances. The horses also performed more standing rest when housed in the single boxes than in the lying halls. Since the lying halls were the only area in the open barns to be video recorded, it is possible that the horses performed resting behaviors outside the lying halls. Some horses could have performed standing rest outside when they only had access to the smaller lying area of 8 m^2^/horse, due to competition for space. In addition, they would have had a better environmental overview outside the lying hall. Another factor that could have increased the lying time in the single boxes was that the bedding in the single boxes was shavings, which may have led to increased lying time as the horses were not inclined to forage from their straw bedding as they were in the lying halls.

Both the sternal and the lateral recumbency in the lying halls increased when the horses were offered 18 m^2^ available lying area instead of 8 m^2^ available lying area. This result is consistent with Fader & Sambraus [21] and Raabymagle & Ladewig [23], who also observed longer lying times in larger lying areas than in smaller ones. Fader & Sambraus [21] found no correlation between number of lying halls and different lying times in their study of seven heterogeneous herds. This indicates that it is in fact the differences in available lying area which affect lying time, and not the separated lying areas when the lying area increased in Study 2 and could be due to the access to greater individual space afforded by larger lying areas. Nevertheless, estimated average lying times when the horses only had access to a lying area of 8 m^2^/horse were no shorter than the lying times observed among feral horses [8,10,11]. The five focal horses in Study 1 seemed to spend less time in lateral and sternal recumbency when comparing the time budgets between the two studies. This finding is difficult to analyze, since lying time has been found to differ between individual horses [19]. Access to a comfortable and secure lying area might be a resource that horses compete for [19,20,21]. Decreased lying time could be associated with inappropriate environmental conditions [7], equating to a limited resource which could, in turn, impact their welfare. Total resting time, including sternal and lateral recumbency and standing rest, did not differ between different available lying areas in lying halls.

In both studies, the horses used the lying halls for purposes other than resting, such as standing attentive, which in Study 1 included foraging. Foraging was therefore separated from standing attentive in Study 2, where foraging from the straw bedding was shown to increase with increased lying area. Straw bedding in lying halls might decrease lying time when compared with other soft bedding due to disturbances caused by foraging [27]. Such disturbances could explain the decrease in lying time in the smaller available lying areas. One advantage of straw bedding is that the horse-keeper can see if the horse has been lying down without video recording, and another is that straw has a positive environmental impact, if it is to be included in an ecological cycle. Offering straw outside the lying hall could be a solution to satisfy the horses’ eating requirements. In both the open barn systems, the horses were offered haylage from automatic, computer-controlled forage stations outside the lying halls, to meet the recommended requirement of 1.5 kg DM per 100 kg bodyweight. The forage feed intake rate is, on average, 22 min/kg DM [34], which means that their estimated daily forage intake time is 3.5 h and suggests that straw is required to meet their need for long feeding times. 

The data from this study are based on observations from two different active open barns with similar stable furnishings, such as lying halls with straw, gravel paddocks, and individual computer-controlled feeding stations. The layouts did differ, however, between the open barns, which may have influenced the lying times that were observed. Furthermore, geographical differences affecting daylight and climate may also have influenced the horses’ behavior, since the open barn used in Study 2 lies 600 km north of the open barn used in Study 1. The horses in both studies were all adult geldings and horses working on a medium level, which means that the horses in the two herds were comparable. Given the similarities between the barns and the horses, the results are equivalent and suggest that there is an optimal lying area per horse in the lying hall. However, it is also important to remember that this optimal lying area could differ in herds with mixed sexes, or herds comprising only mares, as they have different individual spacing needs [34]. In this study, we had no evaluation of the ranks of the individual horses, which has also been reported to influence lying time [20]. Using straw as the bedding material might have influenced the optimal lying area, since straw has been shown to decrease lying time, due to foraging [27]. Another factor pertaining to bedding that could influence lying time is the horses’ prior experience with different bedding materials, which we had no record of.

The observations made through video recordings gave a good overview of the horses’ behaviors, and our results are based on quite a large sample. Although it can be difficult to see if time and treatment covariate, each horse in Study 2 acted as their own control, which lends credibility to our findings. Only five focal horses were used for the interval observations of Study 1, and although these were chosen randomly and were of representative age (10–16 years) and daily work levels, using another five horses may have affected the result due to individual differences. When using scan sampling, there is a possibility of missing some details, such as the duration between the logging of each observation and behaviors exhibited in those intervals. It is hoped, however, that these disadvantages are compensated for by the ability to analyze more animals in less time. In Study 2 there is missing data for one 24 h period in which the horses had access to a lying area of 18 m^2^/horse, due to a demolished fence between the lying hall and a second lying hall that was not in use. The statistics model used compensates for the missing data, meaning that the result is still valid.

The active barn system is suitable for urban horse-keeping and for riding schools with limited access to land for paddocks and grazing and meets all the horse’s basic needs. Overall, however, our observations suggest that the current minimum lying area requirements (at least those stipulated by Swedish legislation) might be too small for the domesticated horse, and that the minimum requirement could be increased in order to better safeguard their welfare. Our recommendation, in order to improve the welfare of horses kept in open barns, is to increase the minimum requirement of the lying area by a factor of 20–100%. This recommendation is based on the finding that to offer a lying area extended by only 20% (Lying hall 2 in Study 2) encouraged the horses to prefer that specific lying hall. Furthermore, increasing the available lying area increased both the use of the lying area and the lying time. Since it is crucial to the welfare of horses that they have the opportunity to get enough sleep, the choice of bedding is an important factor to consider. However, further studies are needed to ascertain the optimal lying area per horse and bedding material in a lying hall, in order to maximize the sleeping comfort of each individual.

## 5. Conclusions

In this study conducted in Sweden in two active open barns, we found that the horses’ lying time increased when they were given access to an individual space in a single box or a larger common space. Increasing the area in the lying hall also increased the use of the lying hall. The hypothesis that increasing the lying area will increase the horses’ total lying time was accepted. 

## Figures and Tables

**Table 1:** Ethogram of behaviors exhibited and recorded during video observation in Study 1. Most behaviors were the same in Study 2, but some were divided into two categories.

Behavior Exhibited	Study 1	Study 2
Sternal recumbency	Lying down on breast with head up or nose on bedding, legs under or next to the body	
Lateral recumbency	Lying down on side with body and head on bedding, legs stretched out	
Standing rest	Standing in relaxed state with head lowered/ relaxed ears/eyes closed/one rear foot slightly elevated	
Standing attentive	Standing with open eyes/ears pointed forward /head upright or eating	Standing with open eyes/ears pointed forward/head upright
Foraging		Standing with nose in bedding or eating in the single box or the lying hall
Active	In motion (including walking, playing, urinating)	
Walk		Walking
Other		Playing, urinating, human interaction (i.e., horse being tied up for mucking out)
Not in lying hall	Outside the lying hall with whole body or only the head	
Not in active open barn/box		Removed from active open barn or individual box

## Data Availability

Data is contained within the Supplementary Material. The data presented in this study are available in https://www.mdpi.com/article/10.3390/ani11113214/s1, Table S1: Rawdata Study 1—Rec. obs. focal horses, No of horses in lying hall, Table S2: Rawdata Study 2—Recorded obs. per horse, No horses in lying hall.

## Supplementary Materials

The following are available online at https://www.mdpi.com/article/10.3390/ani11113214/s1, Table S1: Rawdata Study 1—Rec. obs. focal horses, No of horses in lying hall, Table S2: Rawdata Study 2—Recorded obs. per horse, No horses in lying hall.
